# Transcriptome Profiling Associated with Carcass Quality of Loin Muscles in Crossbred Pigs

**DOI:** 10.3390/ani10081279

**Published:** 2020-07-27

**Authors:** Sang-Mo Kim, Kesavan Markkandan, Jong-Young Lee, Gye-Woong Kim, Jae Young Yoo

**Affiliations:** 1 Department of Animal Resources Science, College of Industrial Sciences, Kongju National University, Yesan-eup, Yesan-gun, Chungcheongnam-do 32588, Korea; cellksm@naver.com; 2 Oneomics Co Ltd., Bucheon-si, Gyeonggi-do 14585, Korea; kesavan@oneomics.co.kr (K.M.); jylee@oneomics.co.kr (J.-Y.L.); 3 Viral Disease Research Division, Animal and Plant Quarantine Agency, Ministry of Agriculture, Food and Rural Affairs, Gimcheon-si, Gyeongsangbuk-do 39660, Korea

**Keywords:** differentially expressed genes, meat quality, RNA sequencing, Z-disc, apelin signaling pathway

## Abstract

**Simple Summary:**

Carcass quality traits, such as lean depth and loin depth, are of extreme economic importance for the swine industry. This study aimed to identify the gene expression pattern related to carcass quality in crossbred pigs ((Landrace × Yorkshire) × Duroc). In total, 20 crossbred pigs were used in this study and divided into two groups based on the loin muscle quality grade. Total RNA samples extracted from the loin muscles of both groups were submitted for RNA-seq. Differentially expressed gene (DEG) analysis of the two groups revealed 282 up-regulated and 189 down-regulated genes (*p* ≤ 0.01), linked to tissue development, striated muscle tissue development, tissue morphogenesis, and lipid metabolic process gene ontology (GO) terms. Kyoto Encyclopedia of Genes and Genomes (KEGG) enrichment analysis highlighted genes related to the calcium signaling pathway, apelin signaling pathway, and the mammalian target of rapamycin (mTOR) signaling pathway. We constructed an expressed gene catalog, which may serve as a resource for genomic studies focused on uncovering the molecular mechanisms underlying carcass quality in crossbred pigs.

**Abstract:**

Carcass quality traits, such as lean depth and loin depth, are of extreme economic importance for the swine industry. This study aimed to identify the gene expression pattern related to carcass quality in crossbred pigs ((Landrace × Yorkshire) × Duroc). In total, 20 crossbred pigs were used in this study and they were divided into two groups (class I grade, n = 10; class II grade, n = 10) based on the carcass grades. Total RNA samples extracted from the loin muscles of both groups were submitted for RNA-seq. The quality assessment of the sequencing reads resulted in 25,458 unigenes and found 12,795 candidate coding unigenes with homology to other species after annotation. Differentially expressed gene (DEG) analysis of the two groups revealed 282 up-regulated and 189 down-regulated genes (*p* ≤ 0.01), linked to tissue development, striated muscle tissue development, tissue morphogenesis, and lipid metabolic process gene ontology (GO) terms. Kyoto Encyclopedia of Genes and Genomes (KEGG) enrichment analysis highlighted genes related to the calcium signaling pathway, melanogenesis, the sphingolipid signaling pathway, the apelin signaling pathway, and the mTOR signaling pathway. We constructed an expressed gene profile, which may serve as a resource for genomic studies focused on uncovering the molecular mechanisms underlying carcass quality in crossbred pigs.

## 1. Introduction

Pork is the most consumed protein source (12.3 kg/capita/year) worldwide [1]. Generally, meat customers are influenced more by product appearance, such as meat color and marbling, than by any other factor [2,3]. In addition, Korean consumers have a unique consumption pattern and a strong preference for high-fat cuts such as belly, Boston butt, and rib [3]. 

Intramuscular fat (IMF) is consistent with pork marbling, which is an important indicator of meat quality [4]. Calcium ions are also important for muscle contraction and relaxation, as well as porcine stress syndrome [5,6]. These meat characteristics have been used in genetic improvement programs in commercial pig lines [7]. However, they have dramatically altered many porcine muscle characteristics, such as IMF, the loin area, and backfat thickness, and have had negative impacts on palatability measures, such as juiciness, flavor, tenderness, and overall acceptability [7,8].

Traditionally, quantitative trait loci have been identified to accelerate the genetic control of economically important traits by means of various candidate genes, such as the ryanodine receptor (*RYR1*), protein kinase *AMP*-activated non-catalytic subunit gamma 3 (*PRKAG3*), fatty acid-binding protein 3 (*HFABP*), and melanocortin 4 receptor (*MC4R*) [9,10]. Recently, studies have investigated the transcript abundance of a group of candidate genes involved in metabolism, nutrient metabolites, and muscle structural genes [4,7,8,11,12,13,14]. These studies identified key regulatory genes that are significantly associated with pork quality and muscle characteristics in purebred pigs. To our knowledge, these studies did not perform transcriptome analysis to detect changes in gene expression associated with carcass grade in crossbred pigs (Landrace × Yorkshire × Duroc). Our study used RNA sequencing methods to associate differentially expressed genes (DEGs) with loin muscle carcass quality in crossbred pigs.

## 2. Materials and Methods

### 2.1. Animals and Sample Collection

Pork carcasses are graded in terms of loin muscle quality and conformation based on the Korea Institute for Animal Products Quality Evaluation (KAPE) and scored as first plus (1 ^+^), first (1), or second (2), based on marbling, lean color, backfat thickness, and body weight [15]. In Korea, all domestically produced pork is graded, which enables the standardized pork distribution system across the country. Pork is indicated as 1 ^+^, 1, or 2 (primary decision and secondary decision), and the quality of pork belly, which has a high added value, is considered to be the highest. The 1 ^+^ grade pork showed a carcass weight from 83 kg to 90 kg and a backfat thickness from 17 mm to 25 mm. Additionally, pork belly was more than 10.72 kg and the fat rate was from 22% to 42%. Twenty crossbred pigs were determined to be grades 1 ^+^ (class I) and 2 (class II) after being slaughtered according to KAPE standards. The carcass quality tenderloin muscle characteristics are listed in Table 1 and Supplementary Table S1. For transcriptomic analysis, 20 samples of loin muscles were immediately frozen in liquid nitrogen and stored at −80 °C until RNA extraction.

### 2.2. Library Preparation and Data Generation

Total RNA was isolated from the indicated tissues using the Qiagen RNeasy^®^ Mini kit (Qiagen, Valencia, CA, USA), and purification was performed according to the manufacturer’s instructions. The RNA concentration was determined using a NanoDrop ND-1000 spectrometer (Nanodrop technologies Inc., Wilmington, NC, USA), and the RNA integrity number was evaluated on a 2100 Bioanalyzer (Agilent Technologies, Santa Clara, CA, USA) using the RNA 6000 Nano Kit (Agilent Technologies). The mRNA libraries were prepared using the TruSeq Stranded mRNA Sample Preparation Kit (RS-122-2101) (Illumina, San Diego, CA, USA). Oligo (dT)’s attached to magnetic beads were used to purify poly-A-containing mRNA from 1 μg of total RNA. Next, the purified mRNA was disrupted into short fragments, and first-strand cDNAs were synthesized using SuperScript II reverse transcriptase (Invitrogen, Carlsbad, CA, USA) and random hexamers. The cDNA with adapters ligated to both ends were enriched by PCR. The cDNA library size and quality were evaluated electrophoretically using the Agilent DNA 1000 Kit (part # 5067-1504) (Agilent Technologies, Santa Clara, CA, USA) on a 2100 BioAnalyzer. Subsequently, the libraries were sequenced on an Illumina HiSeq 2500 (Illumina, San Diego, CA, USA). Image analysis was performed using the HiSeq control software version 2.2.58 (Illumina, San Diego, CA, USA). Raw data were processed and base calling was performed using the standard Illumina pipeline (CASAVA version 1.8.2 and RTA version 1.18.64, Illumina, San Diego, CA, USA). 

### 2.3. Data Analysis

Total sequenced reads were subjected to preprocessing as follows: adapter trimming was performed using cut adapt with the default parameters, and quality trimming (Q30) was performed using FastQC (Babraham Institute, Cambridge, UK) with the default parameters. Processed reads were mapped to the pig reference genome (*Sus scrofa*—Ensembl 89) using TopHat (ver. 2.0.9) and Cufflinks (ver. 2.1.1) with the default parameters [16]. Fragments per kilobase of exon model per million reads mapped (FPKM) values were normalized and quantitated using the R package Tag Count Comparison to determine statistical significance (e.g., *p* and Q values) and differential expression (e.g., fold changes) [17]. The number of DEGs was analyzed with the number of reads estimated by the Kalisto program [18]. Reads were subjected to functional annotations by a sequence homology search against biological databases such as Gene Ontology (GO), and the Kyoto Encyclopedia of Genes and Genomes (KEGG) pathway [19]. Output files of differentially expressed (DE) genes (False Discovery Rate (FDR)  <  0.05) were used in Gene Set Enrichment Analysis (GSEA) to evaluate the relationship between gene expression patterns significantly associated with carcass quality (Kobas 3: http://kobas.cbi.pku.edu.cn/kobas3/). In this study, we focused only on the quantification of known mRNA sequences. Therefore, unknown genes or isoforms were not considered in this study.

## 3. Results

### 3.1. Pig Loin Muscle Transcriptome Profiling

**Figure 1:**
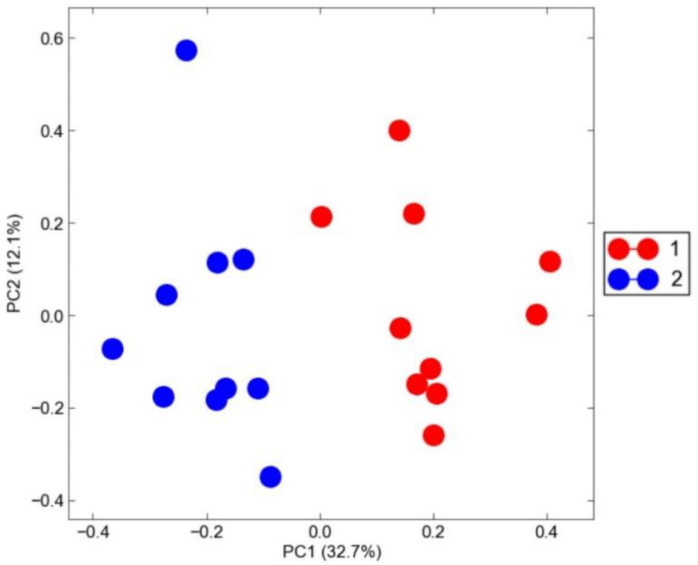
Plot of principal component analysis. Relative abundance log folds accounting for the total raw count are shown. Red point: class I (first plus grade), blue point: class II (second grade).

In this study, twenty cDNA libraries from class I and class II groups were constructed using Illumina sequencing. Principal Component Analysis (PCA) showed that the samples were clustered according to class I and class II (Figure 1). A total of 25,458 clean mapped transcripts were considered for further analysis, and a total of 471 genes were found to be significantly differentially expressed (log_2_FC > 2 and q < 0.01), of which 282 and 189 genes were either up- or down-regulated between class I and class II, respectively (see Supplementary Table S2).

### 3.2. Putative Candidate Genes Involved in Loin Muscle Carcass Quality 

**Figure 2:**
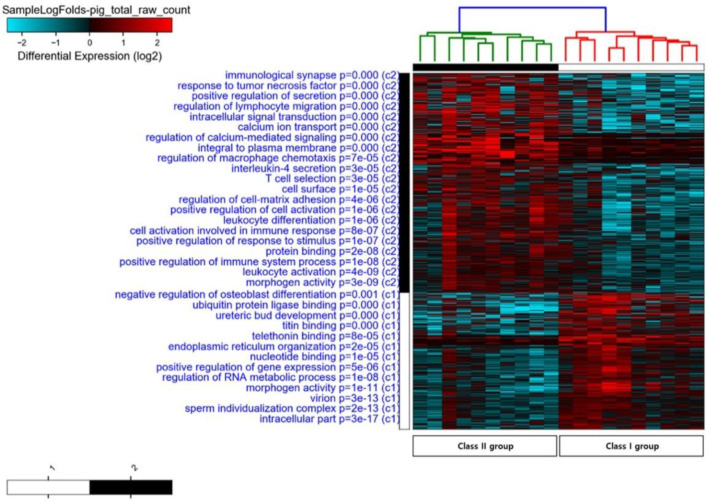
Heatmap gene cluster classification for the class I and class II muscle samples. C1 (white) and C2 (black) refers to the cluster 1 and cluster 2, respectively. Using the expression for each gene (in rows) and sample (in columns), the heatmap was generated by the R package “plots”.

To identify the putative biological function of the DEGs, GO function and KEGG pathway enrichment analysis were performed for the libraries from class I and class II by assigning GO terms associated with the unigenes. Significantly, 2757 gene sets were enriched (FDR < 25%) in class I and class II. DEGs in class II showed that the GO terms of metal ion binding (GO:0046872), cation binding (GO:0043169), immune response cell activation (GO:0006629), cell surface (GO:0009986), calcium ion transport (GO:0006816), tissue development (GO:0009888), striated muscle tissue development (GO:0014706), tissue morphogenesis (GO:0048729), and the cellular lipid metabolic process (GO:0044255) were enriched (See Supplementary Table S3; Figure 2).

Based on previous studies, we filtered out some genes in order to gain a better understanding of the 116 candidate genes and their putative roles in loin muscles (Table 2). Among the GO categories, metal ion transport was enriched, including calcium voltage-gated channel subunit alpha 1 S (*CACNA1S*), calcium/calmodulin-dependent protein kinase II gamma (*CAMK2G*), potassium voltage-gated channel subfamily D member 2 (*KCND2*), potassium voltage-gated channel subfamily Q member 4 (*KCNQ4*), presenilin 1 (*PSEN1*), sodium voltage-gated channel alpha subunit 4 (*SCN4A*), and solute carrier family 9 member A4 (*SLC9A4*). In terms of molecular function, the following genes were predicted to be related to ATP binding: alpha-protein kinase 3 (*ALPK3*), apoptotic protease-activating factor 1 (APAF1), ATPase H+ transporting accessory protein 1 (*ATP6AP1*), *CAMK2G*, dual-specificity tyrosine-phosphorylation-regulated kinase 1B (*DYRK1B*), eukaryotic initiation factor 4A-II (*EIF4A2*), inositol hexakisphosphate kinase 3 (*IP6K3*), nucleotide-binding protein 1 (*NUBP1*), protein kinase domain containing, cytoplasmic (*PKDCC*), proteasome 26S subunit, ATPase 6 (*PSMC6*), RecQ-like helicase 4 (*RECQL4*), structural maintenance of chromosomes 6 (*SMC6*), striated muscle-enriched protein kinase (*SPEG*), ubiquitin-conjugating enzyme E2 D3 (*UBE2D3*), and unc-51-like autophagy activating kinase 1 (*ULK1*). In addition, genes related to anion binding with *ALPK3*, *APAF1*, *ATP6AP1*, *CAMK2G*, *DYRK1B*, early endosome antigen 1 (*EEA1*), *EIF4A2*, fatty acid-binding protein 4 (*FABP4*), GUF1 homolog, GTPase (*GUF1*), *IP6K3*, NLR family pyrin domain containing 1 (*NLRP1*), *NUBP1*, *PKDCC*, *PSMC6*, *RECQL4*, *SMC6*, sorting nexin 13 (*SNX13*), *SPEG*, *UBE2D3*, *ULK1*, and UDP-glucuronate decarboxylase 1 (*UXS1*) genes were found. Significantly, integrin subunit beta 1 binding protein 2 (*ITGB1BP2*), myotilin (*MYOT*), myozenin 3 (*MYOZ3*), protein phosphatase 2 regulatory subunit B’alpha (*PPP2R5A*), and *PSEN1* were classified as Z-disk-related in the cellular component category (false discovery rate = 0.0153). Furthermore, the cation channel complex was associated with *CACNA1S*, *KCNQ4*, and *SCN4A* in the cellular component category.

In the case of the KEGG enrichment pathways, we mapped the DEGs into the KEGG database to search for genes involved in signaling pathways. A total of 471 DEGs were assigned to 240 KEGG pathways (see Supplementary Table S4). Some of the significantly enriched KEGG pathways were related to the signaling pathways, including the cyclic adenosine monophosphate (cAMP) signaling pathway, mitogen-activated protein kinase (MAPK) signaling pathway, calcium signaling pathway, AMP-activated protein kinase (AMPK) signaling pathway, mammalian target of rapamycin (mTOR) signaling pathway, sphingolipid signaling pathway, and apelin signaling pathway. Moreover, there were candidate genes involved in pathways related to glycine, serine, and threonine metabolism, autophagy-animal, and melanogenesis.

## 4. Discussion

In this study, we determined the DEGs associated with carcass grade through transcriptome sequencing in crossbred pigs. We identified associations of the Z-disc, cation channel complex, ion transmembrane transport, protein tyrosine kinase activity, metal ion transport, and apelin signaling pathway with carcass grade using RNA sequencing. The Z-disk is a simple structure at the lateral border of the sarcomere unit in muscles that plays a role in the structure, maintenance, and protein quality control mechanisms of muscles in humans and animals [20,21,22,23,24]. 

In the present study, we showed that the expression of *ITGB1BP2*, *MYOT*, *MYOZ3*, *PPP2R5A*, and *PSEN1* in muscles differed according to carcass grade. Generally, muscle fiber type is an important factor influencing meat quality, including meat color, tenderness, and water-holding capacity (WHC) [21,25,26]. In addition, the MYOZ family of genes (e.g., *MYOZ3* and *MYOT*) influences the formation and maintenance of the Z-disc, which includes α-actinin, β-filamin, telethonin, and calcineurin [21,23]. The *MYOT* genes are known as the titin immunoglobulin domain (*TTID*) gene and localize to the Z-line in muscles and are associated with various carcass traits, such as loin eye area, meat pH, meat color, value, and WHC [27]. Collectively, these data suggest that the cellular components of the Z-disc have important relationships with genes, such as *MYOZ3* and *MYOT*, affecting carcass grade in crossbred pigs.

We assessed ion channel function, such as the cation channel complex, ion transmembrane transport, and metal ion transport. Genetic approaches have shown that ion channel function is closely related to increased and decreased Ca^2+^ ion sensitivities of muscle contraction [20,22]. Typically, the *RYR1* gene causes uncontrolled release by Ca^2+^ release channels within the sarcoplasmic reticulum in response to stress [10,28]. Additionally, *CACNA1S* and *UBE2D3* genes have been found with DEG analysis in relation to tenderness in pork [7]. Gene expression has been associated with various meat quality traits, as well as the concentration of some minerals, e.g., calcium, copper, iron, potassium, magnesium, manganese, sodium, phosphorus, selenium, and zinc [29]. Thus, we suggest that the expression of genes related to metal ion function (e.g., *CACNA1S* and *UBE2D3*) is likely to be associated with carcass grade and meat quality. 

We also observed a relationship between the mTOR signaling pathway and *DVL2* and *ULK* and the carcass grade of pork. Fatty acid synthesis was reduced, which enhanced fatty acid oxidation by up-regulating hepatic lipogenic gene expression, and this was mediated through the mTOR pathway [30]. Previous studies have suggested that the activation of mTOR signaling plays an important role during the initial conversion of muscle to meat [29,31]. There are similarities between metabolic pathways, including the mTOR pathway and oncogenic signaling; these similarities are therapeutic targets [31,32]. *DVL2* gene expression has also been shown to be up- and down-regulated in adipose tissue and muscles, respectively, under insulin-resistant conditions [33]. Taken together, these data suggest that *DVL2* and the mTOR pathway might have important roles in carcass grade.

We also observed a relationship between the apelin signaling pathway and carcass quality. It is interesting that the apelin signaling pathway associated with increased myofilament sensitivity to Ca^2+^ ions and increased intracellular pH [34,35]. Furthermore, this signaling pathway has been implicated in hypertension, diabetes, and obesity and it has emerged as a novel therapeutic targeting in humans [34]. Thus, the apelin signaling pathway may be related to carcass quality and grade, but this did not approach the molecular level in the loin muscles of pigs.

## 5. Conclusions

Transcriptome sequencing can be used to provide remarkable information about the molecular basis of the economically important traits of an organism. Here, we described crossbred pigs’ loin muscle transcriptomes. The transcriptome data in this study will be a valuable resource for genomic studies focused on uncovering the molecular mechanisms underlying carcass quality in crossbred pigs. Thus, those candidate genes identified in this study might be used as predictive markers for pork carcass quality.

## Figures and Tables

**Table 1:** Basic characteristics of the study subjects by grade.

Item		1 ^+^	2	Total	Statistical Test
Carcass weight (kg)		89.2 ± 2.35	94.00 ± 4.22	91.6 ± 4.13	t = −3.16 **
Backfat thickness (mm)		20.4 ± 2.17	27.2 ± 4.59	23.8 ± 4.94	t = −4.24 ***
pH		5.66 ± 0.06	5.60 ± 0.04	5.61 ± 0.05	t = 0.86 ^NS^
CIE color	L *	55.06 ± 2.35	59.79 ± 1.77	57.42 ± 3.16	t = −5.09 ***
	a *	9.24 ± 1.09	8.15 ± 0.53	8.69 ± 1.00	t = 2.85 **
	b *	3.00 ± 0.66	3.85 ± 0.43	3.42 ± 0.70	t = −3.42 **

Data are means ± SD. ^NS^: not significant, **: *p* < 0.01, ***: *p* < 0.001, CIE: International Commission on Illumination, L *: indicates lightness, a *: the red/green coordinate, b *: the yellow/blue coordinate.

**Table 2:** Functional gene ontology (GO) categories enriched in the crossbred pigs.

Category	Term ID	Term Description	Observed Gene Count	Background Gene Count	FDR	Matching Genes *
Biological Process	GO:0008104	Protein localization	24	1966	0.0471	*ABRA*, ***ADORA1***, ***AP2A1***, ***ATP6AP1***, ***CAMK2G***, ***DVL2***, *GRIP1*, ***ITGAL***, ***LIN7A***, *MSX1*, ***NRXN1***, ***NUBP1***, *PICK1*, ***PITRM1***, ***PKDCC***, ***PSEN1***, ***RAB3IP***, *RPS6*, *RRAGA*, ***SNX13***, ***UBE2D3***, ***ULK1***, ***VAMP8***, ***VPS18***
	GO:0030001	Metal ion transport	7	664	0.0034	***CACNA1S***, ***CAMK2G***, ***KCND2***, ***KCNQ4***, ***PSEN1***, ***SCN4A***, ***SLC9A4***
	GO:0016192	Vesicle-mediated transport	9	1699	0.0137	***ADORA1***, *ADORA2A*, *ALB*, ***EEA1***, *HSP90AB1*, ***LIN7A***, *MAPK3*, *PICK1*, ***VAMP8***
Molecular Function	GO:0005524	ATP binding	22	1462	0.0182	*AKT3*, ***ALPK3***, ***APAF1***, ***ATP6AP1***, ***CAMK2G***, ***DYRK1B***, ***EIF4A2***, *HSP90AB1*, ***IP6K3***, *LIG1*, *MAPK3*, *MTOR*, *NLRP1*, ***NUBP1***, ***PKDCC***, *PRKCA*, ***PSMC6***, ***RECQL4***, ***SMC6***, ***SPEG***, ***UBE2D3***, ***ULK1***
	GO:0005324	Long-chain fatty acid transporter activity	2	6	0.0324	*FABP3*, ***FABP4***
	GO:0005504	Fatty acid binding	3	27	0.0324	*ALB*, *FABP3*, ***FABP4***
	GO:0043168	Anion binding	31	2696	0.0324	*AKT3*, *ALB*, ***ALPK3***, ***APAF1***, ***ATP6AP1***, ***CAMK2G***, ***DYRK1B***, ***EEA1***, ***EIF4A2***, *FABP3*, ***FABP4***, *GOT2*, ***GUF1***, *HSP90AB1*, ***IP6K3***, *LIG1*, *MAPK3*, *MTOR*, ***NLRP1***, ***NUBP1***, ***PKDCC***, *PRKCA*, ***PSMC6***, ***RECQL4***, *RRAGA*, ***SMC6***, ***SNX13***, ***SPEG***, ***UBE2D3***, ***ULK1***, ***UXS1***
	GO:0019901	Protein kinase binding	11	599	0.0414	***AP2A1***, ***DUSP1***, ***DVL2***, *HSP90AB1*, *MTOR*, ***NR3C1***, *PCNA*, *PICK1*, ***PRMT1***, ***PTPRJ***, *RPS6*
	GO:0004674	Protein serine/threonine kinase activity	7	444	0.000000000313	*AKT3*, ***CAMK2G***, ***DYRK1B***, *MAPK3*, *MTOR*, *PRKCA*, ***ULK1***
Cellular Component	GO:0030018	Z disc	5	122	0.0153	***ITGB1BP2***, ***MYOT***, ***MYOZ3***, ***PPP2R5A***, ***PSEN1***
	GO:0034703	Cation channel complex	3	206	0.0394	***CACNA1S***, ***KCNQ4***, ***SCN4A***

FDR: False discovery rate, *: Bold text: candidate genes in subjects. *ALPK3*: alpha-protein kinase 3, *APAF1*: apoptotic protease-activating factor 1, *ATP6AP1*: ATPase H+ transporting accessory protein 1, *CACNA1S*: calcium voltage-gated channel subunit alpha 1 S, *CAMK2G*: calcium/calmodulin-dependent protein kinase II gamma, *DYRK1B*: dual-specificity tyrosine-phosphorylation-regulated kinase 1B, *EEA1*: early endosome antigen 1, *EIF4A2*: eukaryotic initiation factor 4A-II, *FABP4*: fatty acid-binding protein 4, *GUF1*: GUF1 homolog, GTPase, *IP6K3*: inositol hexakisphosphate kinase 3, *ITGB1BP2*: integrin subunit beta 1 binding protein 2, *KCND2*: potassium voltage-gated channel subfamily D member 2, *KCNQ4*: potassium voltage-gated channel subfamily Q member 4, *MYOT*: myotilin, *MYOZ3*: myozenin 3, *NLRP1*: NLR family pyrin domain containing 1, *NUBP1*: nucleotide-binding protein 1, *PKDCC*: protein kinase domain containing, cytoplasmic, *PPP2R5A*: protein phosphatase 2 regulatory subunit B’alpha, *PSEN1*: presenilin 1, *PSMC6*: proteasome 26S subunit, ATPase 6, *RECQL4*: RecQ-like helicase 4, *SCN4A*: sodium voltage-gated channel alpha subunit 4, *SLC9A4*: solute carrier family 9 member A4, *SMC6*: structural maintenance of chromosomes 6, *SNX13*: sorting nexin 13, *SPEG*: striated muscle-enriched protein kinase, *UBE2D3*: ubiquitin-conjugating enzyme E2 D3, *ULK1*: unc-51-like autophagy activating kinase 1, and *UXS1*: UDP-glucuronate decarboxylase 1.

## Supplementary Materials

The following are available online at https://www.mdpi.com/2076-2615/10/8/1279/s1, Table S1: Basic characteristics of the study all subjects, Table S2: Summary of gene expression and DEG information from loin muscle transcriptome, Table S3: Summary of enriched GO categories assigned to DEG’s, Table S4: Summary of enriched KEGG pathways assigned to DEG’s.
